# Early Structural Deterioration of a Self-Expanding Transcatheter Aortic Valve Prosthesis Presenting With Severe Aortic Insufficiency: A Case Report

**DOI:** 10.7759/cureus.92971

**Published:** 2025-09-22

**Authors:** Pranali R Dave, Melkon Hacobian

**Affiliations:** 1 Medical School, California University of Science and Medicine, Colton, USA; 2 Cardiology, University of California Los Angeles David Geffen School of Medicine, Los Angeles, USA

**Keywords:** bioprosthetic valve failure, severe aortic regurgitation, structural valve deterioration, tavr (transcatheter aortic valve replacement), tavr valve explantation

## Abstract

Transcatheter aortic valve replacement has become an established therapy for severe aortic stenosis, demonstrating prosthesis durability for at least five years (and up to eight years) in recent studies. Structural valve deterioration within 5 years is rare, particularly with self-expanding Evolut prostheses. We present an 88-year-old man with prior coronary artery bypass grafting and severe aortic stenosis who underwent transfemoral transcatheter aortic valve replacement with a 34 mm Evolut valve in 2019. Five years later, he developed progressive dyspnea and chest pain. Examination revealed a grade IV/VI decrescendo diastolic murmur and a markedly wide pulse pressure, raising concern for severe aortic regurgitation. Transesophageal echocardiography demonstrated moderate to severe aortic insufficiency. Coronary angiography revealed patent grafts, without new obstructive native coronary disease. He underwent surgical aortic valve replacement with an Inspiris Resilia bioprosthesis. This case underscores the possibility of rapid prosthetic valve failure and highlights the ongoing importance of physical examination in detecting severe regurgitation. It also contributes to the growing literature on early valve degeneration.

## Introduction

Transcatheter aortic valve replacement (TAVR) has revolutionized the management of severe aortic stenosis since its introduction in 2002, initially reserved for patients at prohibitive surgical risk and now widely adopted in intermediate- and low-risk populations. Large randomized trials and national registries have demonstrated excellent short- and mid-term outcomes across a broad spectrum of patients. Importantly, valve durability has become a central concern as younger, lower-risk patients undergo TAVR.

The NOTION trial, which randomized low-risk patients to TAVR versus surgical aortic valve replacement (SAVR), reported no significant difference in structural valve deterioration (SVD) at eight years, with rates of approximately three percent in both groups [1]. The SURTAVI trial, enrolling intermediate-risk patients, demonstrated sustained hemodynamic performance and comparable survival at five years with self-expanding valves compared to surgery [2]. The PARTNER 2 and 3 trials, evaluating balloon-expandable prostheses in intermediate- and low-risk patients, similarly confirmed excellent durability and low SVD rates at 5-10 years [3,4]. Large registries, including the STS/ACC Transcatheter Valve Therapy (TVT) Registry in the United States and the FRANCE-TAVI registry, have corroborated these results in real-world populations, showing freedom from SVD exceeding 95 percent at five years [5,6].

Structural valve deterioration refers to intrinsic changes of the valve leaflets or frame that lead to permanent hemodynamic deterioration, whereas bioprosthetic valve dysfunction encompasses SVD and non-structural problems such as thrombosis, pannus, or endocarditis [7]. Valve failure represents the clinical consequence of these processes, manifesting as severe stenosis or regurgitation requiring reintervention.

The declining use and withdrawal of externally mounted surgical bioprostheses (e.g., Trifecta and Mitroflow families) have prompted renewed scrutiny of SAVR durability and prosthesis choice. In parallel, accumulating registry data now describe a “wave” of redo SAVR operations following TAVR SVD, underscoring the growing clinical burden of prosthetic valve failure [8-10]. Reported mechanisms of TAVR SVD include leaflet thickening/calcification, thrombosis, patient-prosthesis mismatch, and endocarditis, with typical time-to-failure beyond five years - though earlier failures have been described. Balloon-expandable TAVR valves are mounted on cobalt-chromium balloon-expandable frames, while self-expanding devices employ nitinol frames; in surgical valves, leaflet mounting may be internal or external, with differing modes of failure.

Against this backdrop, we report rapid degeneration of a large (34 mm) self-expanding nitinol-frame prosthesis, with severe regurgitation occurring within only five years of implantation. This case illustrates the diagnostic value of bedside findings, emphasizes the importance of extended post-procedure surveillance, and discusses management trade-offs between valve-in-valve and surgical explant in this challenging anatomic context.

## Case presentation

An 88-year-old man with a history of coronary artery disease status post coronary artery bypass grafting (2008), hypertension, hyperlipidemia, and antiphospholipid antibody positivity underwent transfemoral TAVR in March 2019 for severe aortic stenosis, receiving a 34 mm self-expanding Evolut prosthesis. His post-procedural course was complicated by a right medial thalamic stroke treated with intravenous thrombolysis, after which he was maintained on dual antiplatelet therapy for six months. Follow-up echocardiography in 2020 and 2022 demonstrated normal prosthesis function.

**Figure 1:**
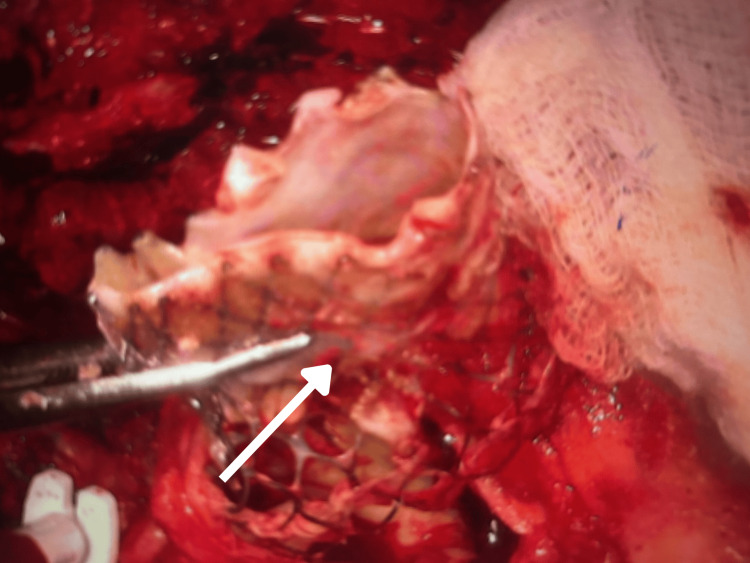
Intraoperative image showing removal of the degenerated 34 mm Evolut transcatheter aortic valve prosthesis.

**Figure 2:**
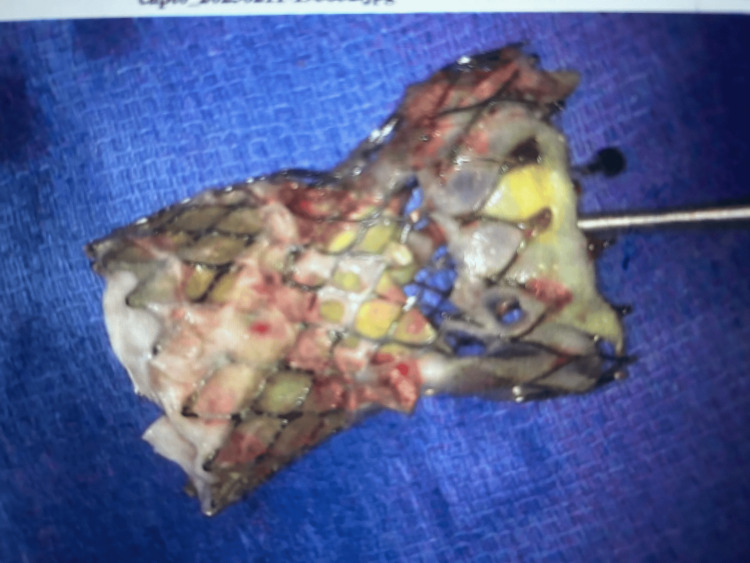
Explanted 34 mm Evolut transcatheter aortic valve prosthesis.

**Figure 3:**
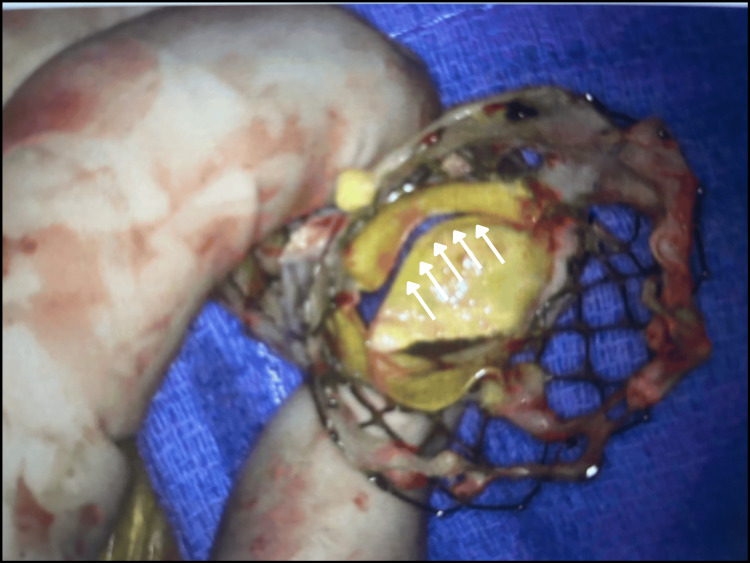
Gross surgical specimen demonstrating markedly abnormal valve architecture, with distorted and non-coapting cusps.

**Figure 4:**
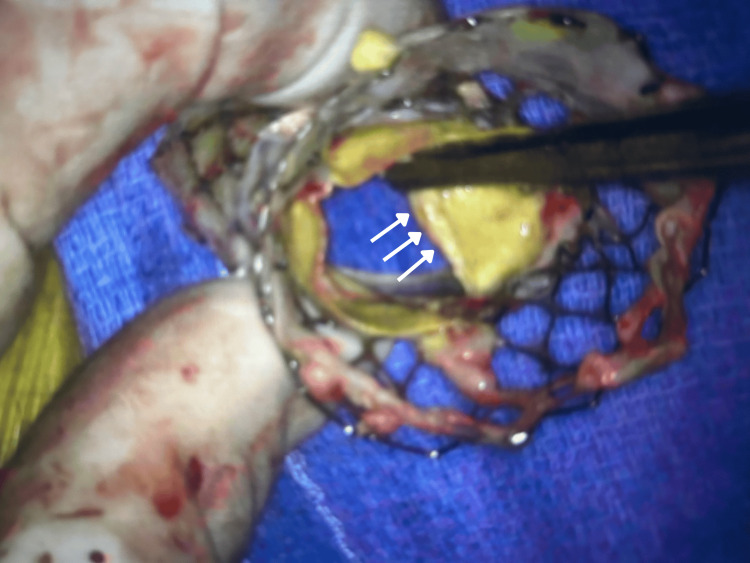
Close-up of the explanted prosthesis, highlighting severe structural deterioration and cusp thickening.

In November 2024, the patient presented with progressive dyspnea and chest pain. Examination revealed a grade IV/VI early diastolic murmur and a markedly wide pulse pressure. Troponin was modestly elevated (peak 444 ng/mL), likely reflecting demand ischemia due to severe aortic regurgitation, as coronary angiography showed no obstructive coronary disease. Transesophageal echocardiography (TEE) revealed moderate to severe aortic insufficiency, while cardiac CT showed no obvious prosthetic complications. Despite optimization of medical therapy, he remained symptomatic, and in February 2025, he underwent redo sternotomy and SAVR with a 23 mm Inspiris Resilia bioprosthesis. Intraoperative inspection confirmed severe structural deterioration of the prior TAVR prosthesis. Postoperative transthoracic echocardiography (TTE) demonstrated an ejection fraction of 36%, with gradual improvement under guideline-directed heart failure therapy (Figures 1-4, Table 1). 

**Table 1:** Imaging Findings Timeline TAVR: Transcatheter aortic valve replacement; TTE: Transthoracic echocardiography; EF: ejection fraction

Date	Modality	Key Findings
Dec 2020	TTE	Normal TAVR prosthesis, preserved EF
Sep 2022	TTE	Normal TAVR prosthesis
Nov 2024 (admission)	TTE	Prosthesis dysfunction suspected
Nov 2024 (hospitalization)	TEE	Moderate–severe aortic insufficiency, no endocarditis
Nov 2024	Cardiac CT	No calcific or structural abnormality
Feb 2025 (post-op)	TTE	EF 36%, functioning surgical bioprosthesis

## Discussion

Registry and trial data suggest that TAVR prostheses provide excellent durability, with outcomes comparable to surgical bioprostheses at 5-10 years [1-6]. SVD remains rare within five years, particularly for self-expanding Evolut valves, with most reports describing freedom from reintervention above 95%. Real-world registry analyses, including the TVT and FRANCE-TAVI registries, corroborate these favorable outcomes, reporting extremely low rates of early degeneration.

Despite these encouraging data, isolated cases of rapid TAVR failure have been described. Mechanisms include leaflet thrombosis, which may be subclinical but associated with hypoattenuated leaflet thickening on CT; accelerated calcification or pannus formation; and structural leaflet injury or dehiscence, often presenting with acute severe aortic regurgitation [7-10]. Our case underscores this last mechanism, with dramatic cusp degeneration leading to severe regurgitation within five years of implantation.

Potential contributors to prosthetic valve deterioration include leaflet dehiscence, cusp avulsion, or pannus overgrowth. Importantly, in this patient, there was no evidence of infection, systemic inflammation, or calcific degeneration to explain the rapid failure. Whether the cusp thickening represented a fibrocalcific versus thrombotic process could not be determined, as histopathologic examination was not obtained.

Crucially, the diagnostic clue in this case was the physical examination. A high-pitched diastolic murmur in conjunction with a strikingly wide pulse pressure remains a classic hallmark of severe aortic regurgitation, even in an era dominated by multimodality imaging. These clinical findings, later corroborated by echocardiography, were pivotal in recognizing early prosthesis failure.

This case also highlights the importance of extended post-procedure surveillance, particularly when patients present with new symptoms such as fatigue, dyspnea, or reduced exercise tolerance. Early recognition of valve dysfunction allows timely intervention and may mitigate morbidity associated with advanced prosthetic failure.

Management of failed TAVR is complex. Valve-in-valve implantation is increasingly utilized, offering reduced early mortality compared with surgical explantation [11]. However, redo SAVR remains essential in cases of severe structural degeneration, cusp tear, or unfavorable anatomy, as in our patient. This highlights the importance of individualized decision-making within the multidisciplinary heart team.

## Conclusions

This case highlights the early structural failure of a self-expanding TAVR prosthesis within only five years, an uncommon but clinically important complication. The physical exam findings of a loud diastolic murmur and wide pulse pressure were the first clear indication of valve dysfunction. Clinicians should maintain vigilance for prosthetic valve degeneration even within expected durability windows and recognize that bedside findings may herald significant pathology. Importantly, this case underscores the enduring value of integrating careful clinical assessment with advanced imaging when evaluating prosthetic valve function. It also serves as a reminder that unexpected complications can arise even with devices considered durable in large clinical trials. Finally, documenting such rare outcomes is essential to guide future patient monitoring and decision-making. Valve-in-valve is often feasible and may carry lower short-term risk in selected anatomies, but surgical explant remains necessary when there is severe leaflet degeneration, unfavorable coronary anatomy, or concern for suboptimal hemodynamics.
